# Fossil Fishes from China Provide First Evidence of Dermal Pelvic Girdles in Osteichthyans

**DOI:** 10.1371/journal.pone.0035103

**Published:** 2012-04-03

**Authors:** Min Zhu, Xiaobo Yu, Brian Choo, Qingming Qu, Liantao Jia, Wenjin Zhao, Tuo Qiao, Jing Lu

**Affiliations:** 1 Key Laboratory of Evolutionary Systematics of Vertebrates, Institute of Vertebrate Paleontology and Paleoanthropology, Chinese Academy of Sciences, Beijing, China; 2 Department of Biological Sciences, Kean University, New Jersey, United States of America; 3 Subdepartment of Evolutionary Organismal Biology, Department of Physiology and Developmental Biology, Uppsala University, Uppsala, Sweden; State Natural History Museum, Germany

## Abstract

**Background:**

The pectoral and pelvic girdles support paired fins and limbs, and have transformed significantly in the diversification of gnathostomes or jawed vertebrates (including osteichthyans, chondrichthyans, acanthodians and placoderms). For instance, changes in the pectoral and pelvic girdles accompanied the transition of fins to limbs as some osteichthyans (a clade that contains the vast majority of vertebrates – bony fishes and tetrapods) ventured from aquatic to terrestrial environments. The fossil record shows that the pectoral girdles of early osteichthyans (e.g., *Lophosteus*, *Andreolepis*, *Psarolepis* and *Guiyu*) retained part of the primitive gnathostome pectoral girdle condition with spines and/or other dermal components. However, very little is known about the condition of the pelvic girdle in the earliest osteichthyans. Living osteichthyans, like chondrichthyans (cartilaginous fishes), have exclusively endoskeletal pelvic girdles, while dermal pelvic girdle components (plates and/or spines) have so far been found only in some extinct placoderms and acanthodians. Consequently, whether the pectoral and pelvic girdles are primitively similar in osteichthyans cannot be adequately evaluated, and phylogeny-based inferences regarding the primitive pelvic girdle condition in osteichthyans cannot be tested against available fossil evidence.

**Methodology/Principal Findings:**

Here we report the first discovery of spine-bearing dermal pelvic girdles in early osteichthyans, based on a new articulated specimen of *Guiyu oneiros* from the Late Ludlow (Silurian) Kuanti Formation, Yunnan, as well as a re-examination of the previously described holotype. We also describe disarticulated pelvic girdles of *Psarolepis romeri* from the Lochkovian (Early Devonian) Xitun Formation, Yunnan, which resemble the previously reported pectoral girdles in having integrated dermal and endoskeletal components with polybasal fin articulation.

**Conclusions/Significance:**

The new findings reveal hitherto unknown similarity in pectoral and pelvic girdles among early osteichthyans, and provide critical information for studying the evolution of pelvic girdles in osteichthyans and other gnathostomes.

## Introduction

The gnathostomes or jawed vertebrates comprise the extant osteichthyans (bony fishes and tetrapods) and chondrichthyans (cartilaginous fishes) along with the extinct placoderms and acanthodians [1]. Girdle-supported paired fins and limbs characterize all jawed vertebrates, and have undergone significant transformation in the course of gnathostome diversification. The pectoral girdles of gnathostomes primitively combine dermal and endoskeletal elements, as in jawless osteostracans [1], [2], [3], [4] even though the osteostracan pectoral girdles are fused to the cranium. For instance, the pectoral girdle in crown osteichthyans (actinopterygians and sarcopterygians) has an endoskeletal scapulocoracoid attached to the inner surface of the cleithrum (one of the encircling dermal bones of the pectoral girdle). However, the primitive condition for pelvic girdles is less clear, resulting from the scarcity of articulated early gnathostome postcrania and the absence of girdle-supported pelvic fins in all known jawless fishes [5]. Both living osteichthyans and chondrichthyans have exclusively endoskeletal pelvic girdles [6]. Until recently, the presence of pelvic girdles with substantial dermal components (large dermal plates) was thought to be restricted to some placoderms (arthrodires, ptyctodonts, acanthothoracids and antiarchs) [7], [8], [9], [10] while pelvic fin spines alone were found in some acanthodians [1], [11]. The purported monophyly of both of these fossil gnathostome ‘classes’ is currently under scrutiny, with most recent phylogenies assigning some or all acanthodians to the osteichthyan stem [1], [4], [12], [13], [14], while resolving the placoderms (either as a monophyletic group or as a paraphyletic assemblage) [1], [10], [12], [15], [16], [17] at the base of the jawed vertebrate radiation. Inferences from these phylogenies would predict that stem osteichthyans more crownward than *Acanthodes* [12], [14] should have at most the pelvic girdles similar to those in acanthodians (i.e., an endoskeletal girdle with a dermal fin spine). Until now, the earliest osteichthyan materials [18], [19], [20], [21], [22], [23] have yielded very little information regarding the primitive condition of pelvic girdles among osteichthyans, making it difficult to test phylogeny-based inferences against the known fossil record or to explore how and when the living osteichthyans may have acquired their exclusively endoskeletal pelvic girdles.

As the first known occurrence in any osteichthyans, here we describe pelvic girdles with substantial dermal components (plates and spines) in two early bony fishes, *Guiyu oneiros* [20], [24] and *Psarolepis romeri* [18], [25], [26], [27], from Yunnan, China. *Guiyu* and *Psarolepis* have been placed as stem sarcopterygians in earlier studies [14], [20], [21], [28], [29], [30], even though they manifested combinations of features found in both sarcopterygians and actinopterygians (e.g. pectoral girdle structures, the cheek and operculo-gular bone pattern, and scale articulation). When *Guiyu* was first described [20] based on an exceptionally well-preserved holotype specimen, it also revealed a combination of osteichthyan and non-osteichthyan features, including spine-bearing pectoral girdles and spine-bearing median dorsal plates found in non-osteichthyan gnathostomes as well as cranial morphology and derived macromeric squamation found in crown osteichthyans. In addition, *Guiyu* provided strong corroboration for the attempted restoration of *Psarolepis romeri* [18], [31] based on disarticulated cranial, cheek plate, shoulder girdle and scale materials [27], [32]. The incongruent distribution of *Guiyu* and *Psarolepis* features across different groups (actinopteryians vs sarcopterygians, osteichthyans vs non-osteichthyans) poses special challenges to attempts at polarizing the plesiomorphic osteichthyan and gnathostome characters and reconstructing osteichthyan morphotype [33], [34], [35]. The phylogenetic analysis in Zhu et al. [18] assigned two possible positions for *Psarolepis*, either as a stem sarcopterygian or as a stem osteichthyan. Basden et al. [21] suggested that *Psarolepis* is more likely a stem sarcopterygian based on the comparison of braincase morphology with an actinopterygian-like osteichthyan *Ligulalepis*. The phylogenetic analysis in Zhu et al. [20] placed *Guiyu* in a cluster with *Psarolepis* and *Achoania* [28] as stem sarcopterygians, with *Meemannia* [23] and *Ligulalepis* [21], [36], [37] as more basal sarcopterygians, and *Andreolepis* [38], [39] and *Lophosteus* [40], [41] as stem osteichthyans.

Although previous studies of *Guiyu* and *Psarolepis* have advanced our understanding of early osteichthyan morphologies beyond what was previously known from *Andreolepis*, *Lophosteus* [19], *Ligulalepis* [21], [37] and *Dialipina* [22], no pelvic girdle components were identified or described at the time, and the primitive condition of pelvic girdles in osteichthyans remained unknown until recently. The situation started to change when a new articulated specimen of *Guiyu oneiros* was collected from the Late Ludlow (Silurian) Kuanti Formation, Yunnan, China. Observations of this new specimen, re-examination of the holotype of *Guiyu oneiros*, and studies of previously unidentified disarticulated specimens of *Psarolepis* form the basis for the finding reported below. As the first evidence for the presence of dermal pelvic girdles in osteichthyans, the pelvic girdles in *Guiyu* and *Psarolepis* reveal an unexpected morphology that stands in stark contrast to the inferences from published phylogenetic analyses (except for one of two alternative positions of *Psarolepis* in Zhu et al. [18]), and appear to resemble those of placoderms [7], [8], [9], [10] rather than either the acanthodians or, indeed, any other previously known osteichthyans.

## Materials and Methods

The specimens are housed at the Institute of Vertebrate Paleontology and Paleoanthropology (IVPP), Chinese Academy of Sciences. The fossil blocks containing the new articulated specimen of *Guiyu oneiros* were collected from the muddy limestone of the Kuanti Formation (Late Ludlow, Silurian), while the disarticulated specimens of *Psarolepis romeri* came from the muddy limestone of the Xitun Formation (Lochkovian, Early Devonian) in Qujing, Yunnan, China [42]. The specimens were prepared mechanically using pneumatic air scribes and needles under microscopes. Illustrative drawings using Adobe Photoshop were produced to highlight or accentuate some morphological features when they would be difficult to see on photographs alone.

The phylogenetic framework for this study is based on the trees in Zhu et al. [18], [20]. We adopt the grouping of *Guiyu* with *Psarolepis* [20] and the alternative positions of *Psarolepis* (either a stem sarcopterygian or a stem osteichthyan) as our working hypotheses [18].

## Results

### (a) Pelvic girdles and related structures in *Guiyu oneiros*

**Figure 1:**
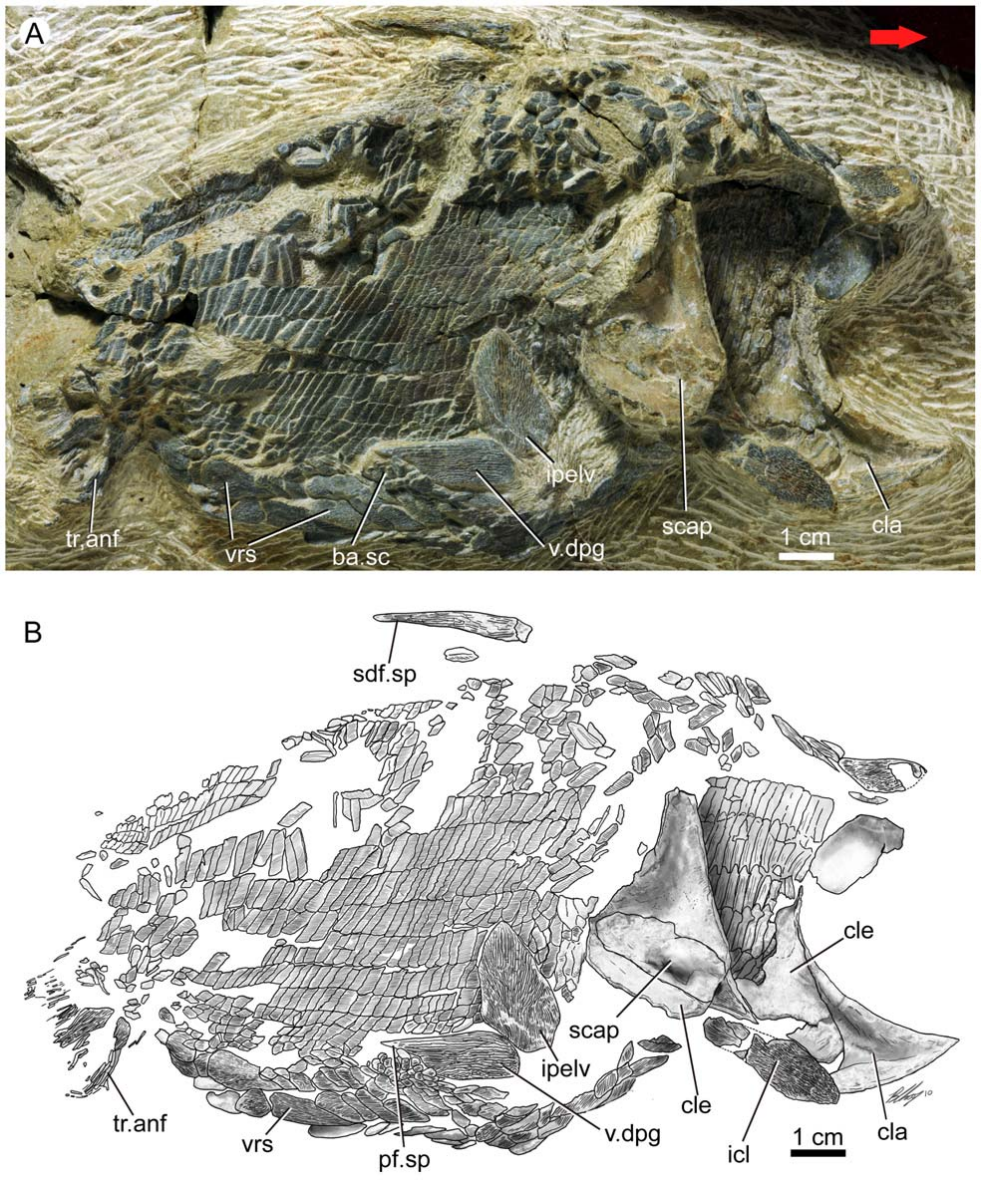
*Guiyu oneiros* Zhu et al., 2009. **A.** New articulated specimen of *Guiyu oneiros* (V17914, lateral view) from the Kuanti Formation (Late Ludlow, Silurian), Qujing, Yunnan, showing a right dermal pelvic girdle in near-natural position. Red arrow points to the anterior end of the fish. **B.** Interpretative drawing. **Abbreviations**: **ba.sc**, basal scales of pelvic fin; **cla**, clavicle; **cle**, cleithrum; **icl**, interclavicle; **ipelv**, interpelvic plate; **pelv.sp**, pelvic fin spine; **scap**, scapulocoracoid; **sdf.sp**, second dorsal fin spine; **tr.anf**, lepidotrichia of anal fin; **v.dpg**, ventral lamina of dermal pelvic girdle; **vrs**, ventral ridge scale.

The new articulated specimen of *Guiyu oneiros* (V17914.1, Fig. 1) was collected in 2010 from the same layer and site as the holotype V15541 [20]. It lacks the skull, however its postcranial preservation is more extensive (extending to the middle level of the anal fin, as inferred from the lepidotrichia, tr.anf, Fig. 1) than that of the holotype. The pectoral girdles are well preserved, comprising both sets of cleithra and clavicles as well as the unpaired rhombic interclavicle (cle, cla, icl, Fig. 1). The massive scapulocoracoid (scap, Fig. 1) is in close contact to the inner side of the cleithrum, as in *Psarolepis* [18], [27].

The most remarkable feature of V17914.1 is the presence of a right pelvic girdle in ventral view (Fig. 1) in a near-natural position, lying more or less exactly ventral to the second dorsal fin spine. The girdle is an oblong bone 17 mm in length (excluding spine) and 7 mm in width with a sharp posterolateral spine (pf.sp, Fig. 1B). The ornament consists of long rostrocaudally directed linear ridges. Immediately adjacent to the spine and the presumed area of fin insertion are a cluster of small rounded scales that, as preserved, lie above the level of the ventral squamation, probably the remains of the fleshy basal lobe of the right pelvic fin (ba.sc, Fig. 1A). No pelvic lepidotrichium is preserved.

**Figure 2:**
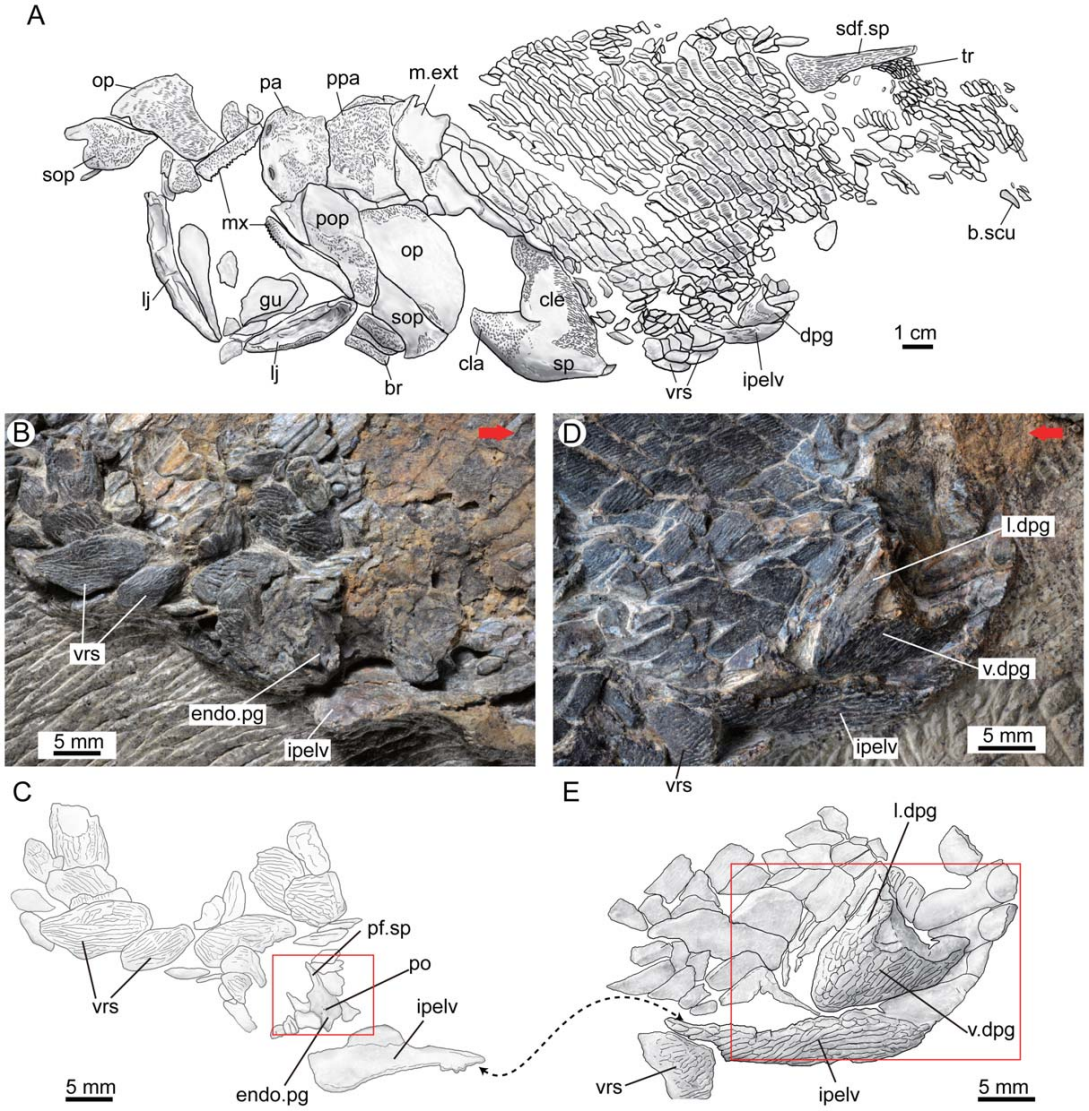
The holotype (V15541) of *Guiyu oneiros* Zhu et al., 2009. **A.** Interpretative drawing of the part to show the position of the newly identified left pelvic girdle with dermal and endoskeletal components. **B**–**C**. Close-up of the counterpart to show the endoskeletal pelvic girdle in internal view (B) and interpretative drawing (C). **D**–**E.** Close-up of the part to show the dermal pelvic girdle in lateral view (D) and interpretative drawing (E). Red arrows point to the anterior end of the fish. The red rectangles indicate the close-up areas in Figure 3A and Figure 3B. The double arrows point to the corresponding positions of the fractured interpelvic plate in part (E) and counterpart (C). **Abbreviations**: **br**, branchiostegal ray; **b.scu**, basal scute; **cla**, clavicle; **cle**, cleithrum; **dpg**, dermal pelvic girdle; **endo.pg**, endoskeletal pelvic girdle; **gu**, gular; **ipelv**, interpelvic plate; **l.dpg**, lateral lamina of dermal pelvic girdle; **lj**, lower jaw; **m.ext**, median extrascapular; **mx**, maxillary; **op**, opercular; **pa**, parietal shield; **pf.sp**, pelvic fin spine; **po**, foramina for pterygial nerves and vessels; **pop**, preopercular; **ppa**, postparietal shield; **sdf.sp**, second dorsal fin spine; **sop**, subopercular; **sp**, pectoral fin spine; **tr**, lepidotrichia; **v.dpg**, ventral lamina of dermal pelvic girdle; **vrs**, ventral ridge scale.

**Figure 3:**
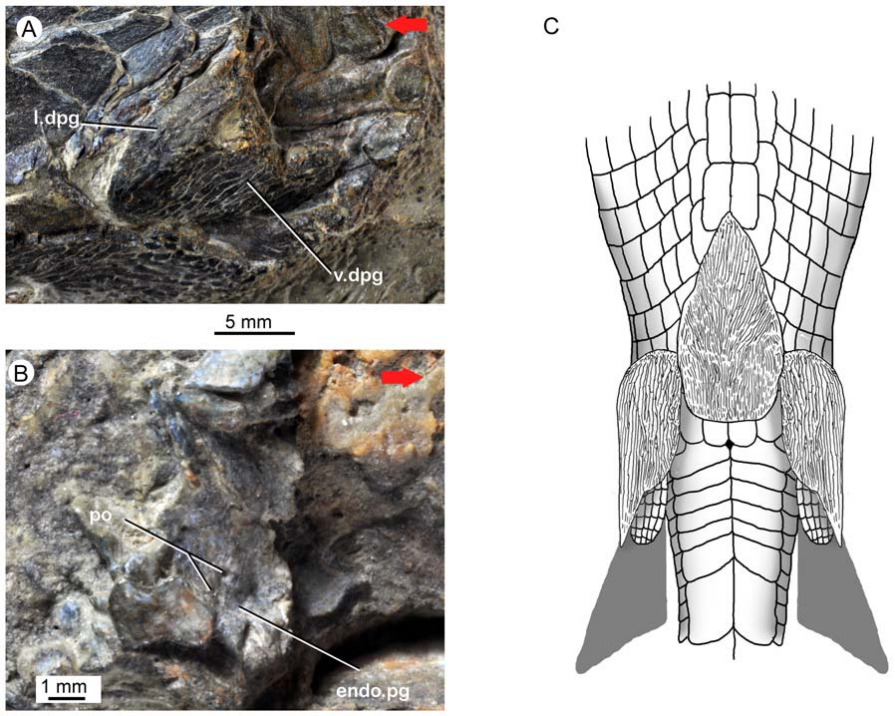
*Guiyu oneiros* Zhu et al., 2009. **A.** Close-up of the holotype (in part) to show the dermal pelvic girdle in lateral view. **B.** Close-up of the holotype (in counterpart) to show the endoskeletal pelvic girdle in internal view. **C.** Tentative life restoration in ventral view to show the paired pelvic girdles and unpaired interpelvic plate. Red arrows point to the anterior end of the fish. **Abbreviations: endo.pg**, endoskeletal pelvic girdle; **l.dpg**, lateral lamina of dermal pelvic girdle; **po**, foramina for pterygial nerves and vessels; **v.dpg**, ventral lamina of dermal pelvic girdle.

Re-examination of the holotype V15541 of *Guiyu oneiros* [20] (Figs. 2, 3) reveals a similarly positioned left pelvic girdle, previously labeled as one of two ventral ridge scales [20]. The anterior half (Figs. 2D, E, 3A), on the part, shows the ventral lamina with its thickened lateral rim curving dorsally to meet the lateral lamina. The posterior half (Figs. 2B, C, 3B), on the counterpart, shows the internal view of the perichondrally ossified endoskeletal girdle with foramina for nerves and vessels (po, Figs. 2C, 3B), resembling the massive scapulocoracoid (scap, Fig. 1) attached to the inner face of the cleithrum in V17914.1.

Preserved immediately anterior to the pelvic girdle of V17914.1 is a large plate-like structure henceforth referred to as the interpelvic plate (ipelv, Fig. 1), which might be considered as a serial homologue of the interclavicle. An identical structure in the holotype is broken into the part (ipelv, Fig. 2A, D, E) and counterpart (ipelv, Fig. 2B, C) but reveals the original position of this plate on the median ventral surface, separating the two pelvic girdles and positioned anterior to the presumed location of the cloaca. In V17914.1, the slightly displaced interpelvic plate is a large lanceolate element over 25 mm in length. It broadens posteriorly and tapers towards an anterior apex with a small raised ridge marking the midline of the unit. Ornament consists of parallel anteroposteriorly-running ridges on the midline and the posterior 1/3 of the plate, with diverging anterolaterally-running ridges on the remainder.

As shown in V17914.1 and the holotype, a greater part of the median ventral surface of *Guiyu* is covered by paired scutes or ridge scales with ganoine striations. Between the pectoral and pelvic regions were more than six pairs of oval scutes whose original orientation is difficult to discern due to post-mortem disruption. Their ventral position and paired nature raise the possibility of homology with the intermediate spines of some acanthodians [43]. Posterior to the interpelvic plate lie a series of small scales that probably framed the cloacal opening. Immediately posterior to these small scales are six pairs of scutes arranged with the long axis directed posterolaterally. The anterior five pairs are narrow with the long axis being about 3.5 times longer than the short axis. The final pair is broader, with the long axis about twice the length of the short axis. Between these scutes and the anal fin are at least two pairs of large, flat plates that are once again arranged with a rostrocaudally oriented long axis.

### (b) Revised restoration of *Guiyu oneiros*

**Figure 4:**
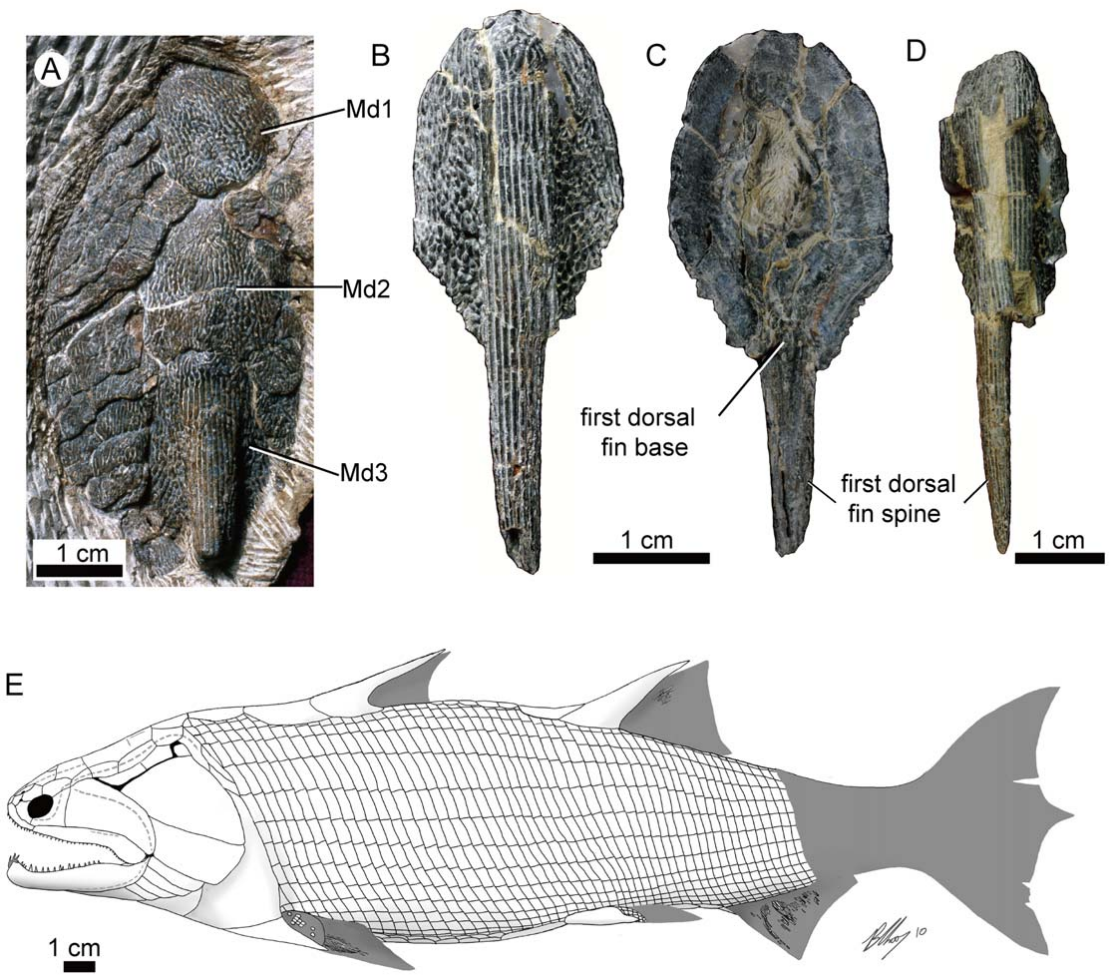
*Guiyu oneiros* Zhu et al., 2009. **A.** Close-up of the three median dorsal plates (Md1-Md3) of the holotype V15541. **B**–**C.** A disarticulated third median dorsal plate (Md3) bearing the first dorsal fin spine in external (B) and internal (C) views, V17914.2. **D.** A disarticulated third median dorsal plate (Md3) in external view, V17914.3. **E.** Revised restoration of *Guiyu oneiros* in lateral view, based on [24] for the cranial portion, and new data in this work. **Abbreviations: Md1**–**Md3**, first to third median dorsal plates, with the third bearing the first dorsal fin spine and the endoskelatal basal plate.

In addition to the presence of dermal pelvic girdles, further modifications are made to the lateral reconstruction of *Guiyu oneiros* (Fig. 4D contra fig. 3a in [20]) based on examinations of the articulated specimens (both V17914.1 and the holotype) and additional disarticulated specimens from the Kuanti Formation, Yunnan.

The premaxillae are more extensive than previously reconstructed, posteriorly terminating beneath the orbit [24]. A series of three (rather than two) median dorsal plates are present behind the median extrascapular (Md1–3, Fig. 4A). The third plate bears the first dorsal fin spine, which, based on disarticulated specimens (Fig. 4B–D), is far more elongate (comparable in length with the second dorsal fin spine) than previously reconstructed [20]. A massive endoskeletal basal plate (Fig. 4C) is attached to the ventral side of the third median dorsal plate. The presence of a first dorsal fin is inferred by the endoskeletal basal plate and a shallow posterior groove on the spine along with the absence of ridge scales in the median dorsal region, which extends about 2–3 cm behind the spine-bearing plate and possibly demarcates the basal extent of the fin.

**Figure 5:**
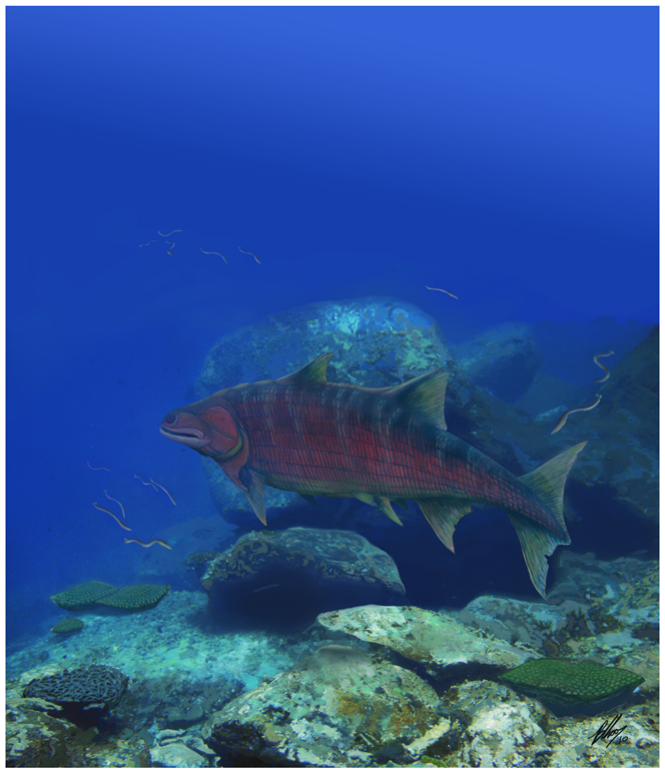
Life restoration of *Guiyu oneiros* from the Kuanti Formation (Late Ludlow, Silurian), Qujing, Yunnan, China.

The anal fin was initially reconstructed as being located directly opposite to the dorsal fin. Based on the extent of the ventral post-pelvic squamation and the position of lepidotrichia in V17914.1 (tr.anf, Fig. 1), it is clear that the fin was far more posteriorly positioned. A basal scute or fulcrum, initially reconstructed as lying at the base of the lower hypochordal lobe of the holotype, is now reinterpreted as marking the anterior margin of the anal fin. While Zhu et al. [20] suggests that the holotype is completely preserved all the way to the caudal peduncle, the actually preserved portion of the specimen terminates at the anterior margin of the anal fin, indicating a more elongate profile (Fig. 5) than previously reconstructed [20]. V17914.1 preserves a slightly longer posterior division, but its anatomy posterior of the anal fin, including the caudal fin, remains unknown.

### (c) Pelvic girdles in *Psarolepis romeri*

**Figure 6:**
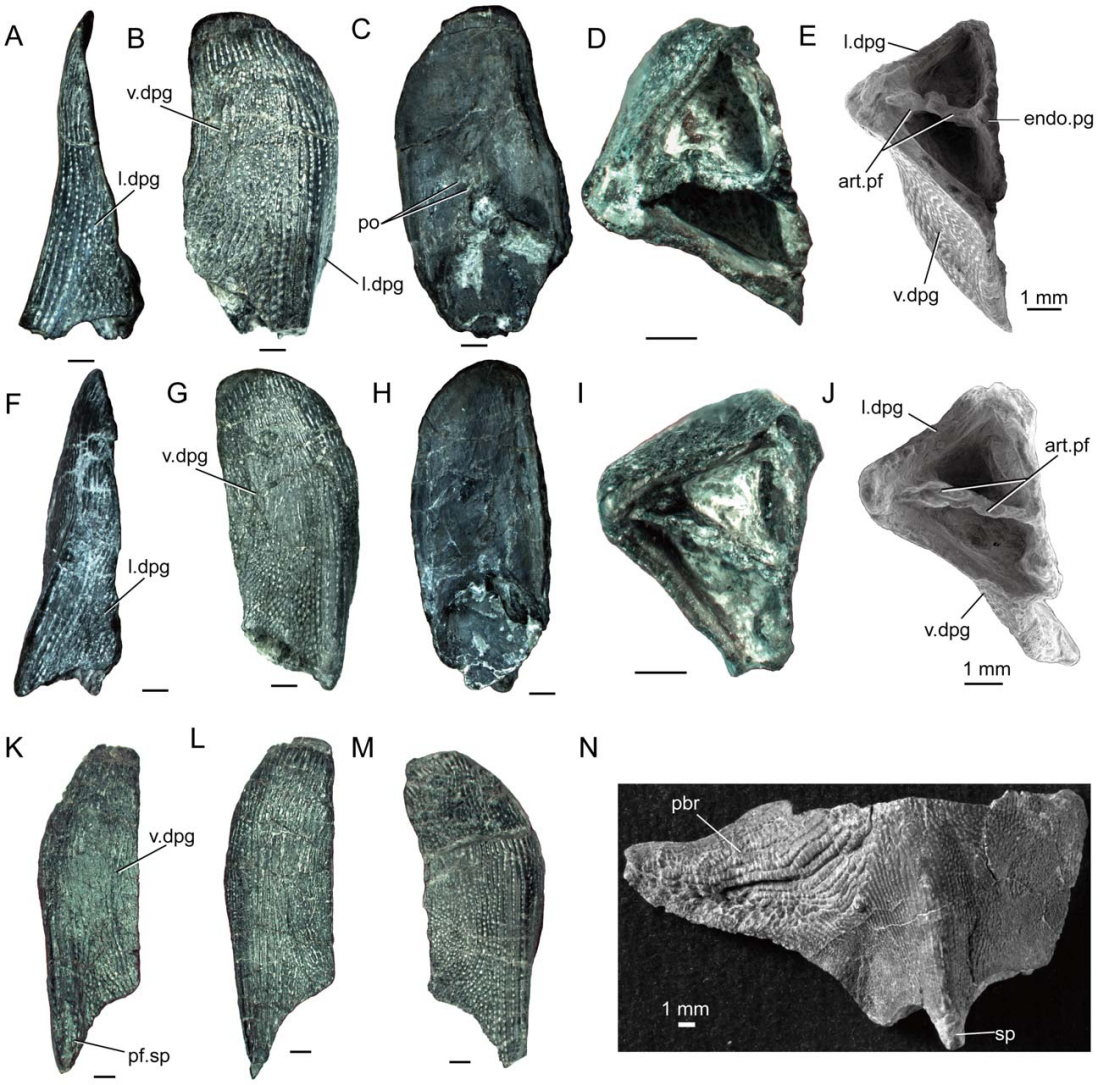
*Psarolepis romeri* Yu, 1998, from the Lower Devonian Xitun Formation (Lochkovian) of Qujing, Yunnan. **A**–**E.** Disarticulated left pelvic girdle in lateral (A), ventral (B), internal (C) and posterior (D–E) views, V17913.2. **F**–**J.** Disarticulated left pelvic girdle in lateral (F), ventral (G), internal (H) and posterior (I–J) views, V17913.1. **K.** Disarticulated right pelvic girdle in ventral view, V17913.5. L. Disarticulated right pelvic girdle in ventral view, V17913.4. **M.** Disarticulated left pelvic girdle in ventral view, V17913.3. **N.** Disarticulated left pectoral girdle in ventrolateral view, V15544.1. E and J are SEM photos. All scale bars equal 1 mm. **Abbreviations: art.pf**, articulation facet for pelvic fin; **endo.pg**, endoskeletal pelvic girdle; **l.dpg**, lateral lamina of dermal pelvic girdle; **pbr**, postbranchial lamina; **pf.sp**, pelvic fin spine; **po**, foramina for pterygial nerves and vessels; **sp**, pectoral fin spine; **v.dpg**, ventral lamina of dermal pelvic girdle.

The presence of pelvic girdles found in two articulated specimens of *Guiyu oneiros* enables the identification of similarly shaped disarticulated elements (V17913.1 to V17913.5; Fig. 6A–M) from Yunnan as the pelvic girdles of *Psarolepis romeri* [18], [26], [27], [32]. These specimens possess the same distinctive large-pored cosmine surface and come from the same beds and locality (Xitun Formation, early Lochkovian) as previously reported materials of *P. romeri* [18], [26], [27], [32].

The dermal pelvic girdle of *Psarolepis* has a profile somewhat similar to that of the cleithrum [27], though its lateral and ventral laminae (l.dpg, v.dpg, Fig. 6A–M) are obviously less extensive than those of the cleithrum. The dermal pelvic girdle presents an elongate rhomboid profile in ventral view with a short posterolaterally extending spine (pf.sp, Fig. 6K). Unlike the cleithrum whose lateral lamina has a particular portion with organized pyramid-like denticles or the postbranchial lamina (pbr, Fig. 6N), the lateral lamina of the dermal pelvic girdle (l.dpg, Fig. 6A, F) is very low anteriorly and lacks any portion similar to the postbranchial lamina.

The perichondrally ossified endoskeletal pelvic girdle (endo.pg, Fig. 6C–E, H–J) is closely attached to the inner face of the dermal girdle, a condition seen in *Guiyu* (Figs. 2B, C, 3B), but also in some placoderms (ptyctodonts and acanthothoracids) [8], [9]. In transverse cross-sectional perspective, the bone with integrated dermal and endoskeletal elements is three-sided with porous cosmine-ornamented ventral and lateral laminae and a smooth visceral face, pierced by several openings for nerves and blood vessels (po, Fig. 6C, H).

In posterior view, the fossa from which the pelvic fin originated is dissected by a horizontal articular crest carrying at least two well defined facets of similar size (art.pf, Fig. 6D, E, I, J). This suggests that *Psarolepis* (and possibly *Guiyu* – by inference) had a polybasal pelvic fin articulation, a condition already established in the pectoral fin articulation [27]. The polybasal pelvic fin articulation was previously known only in actinopterygian osteichthyans [44] and non-osteichthyan gnathostomes [1].

## Discussion

The pelvic girdles of *Guiyu oneiros* [20], [24] and *Psarolepis romeri* [18], [25], [26], [27] are striking in their similarity to the pectoral girdles of these taxa [18], [25], [26], [27] and to the pelvic girdles of placoderms. This challenges existing hypotheses regarding early osteichthyan pelvic evolution based on the putative absence of dermal components and the dissimilarity of the pectoral and pelvic anatomy [6]. Until now, osteichthyans were known to have very different pectoral and pelvic girdles (the former with endoskeletal and dermal components while the latter being exclusively endoskeletal) [6]. The new material, coupled with previously reported pectoral girdle findings [27], reveals hitherto unknown similarity in pectoral and pelvic girdles (both featuring a massive endoskeletal girdle integrated with dermal plates, spines, and polybasal fin articulation) in early osteichthyans.

The pelvic girdle finding lengthens the list of observable similarities between placoderms and osteichthyans (e.g., in dermal skull roof bones and pectoral girdles) [18], [45], [46], [47], [48] and accentuates inconsistencies between the early osteichthyan condition and the presumed acanthodian-like ‘stem’ model implicit in prevailing gnathostome phylogenies [1], [4], [12], [13], [14], [15], [16]. The data presented here provide new morphological information for more focused future studies of these and other phylogenetically controversial Silurian–Devonian osteichthyan forms [14], [20].

**Figure 7:**
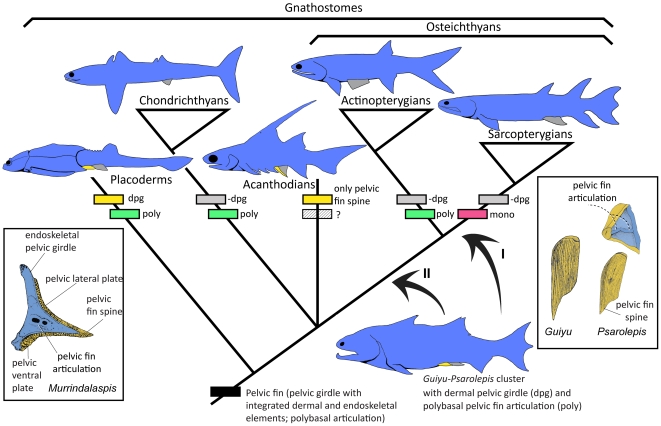
Gnathostome pelvic girdle evolution. The cladogram is based on the trees in Zhu et al. [18], [20]. We adopt the grouping of *Guiyu* with *Psarolepis* [20] and the alternative positions (I and II) of *Psarolepis* (either a stem sarcopterygian or a stem osteichthyan) [18] as our working hypotheses. Drawing of placoderm pelvic girdle (*Murrindalaspis* in posterior view) from [8]. Dermal pelvic girdles (including plates and spines) in yellow; endoskeletal pelvic girdles in blue. **Abbreviations: -dpg**, loss of dermal pelvic girdle; **dpg**, dermal pelvic girdle; **mono**, monobasal pelvic fin articulation; **poly**, polybasal pelvic fin articulation.

Among non-osteichthyan gnathostomes, dermal elements related to the pelvic girdle exist only in some acanthodians (i.e. pelvic spines) and some placoderms (e.g. a single dermal plate in the Ptyctodontida and a three-plated structure, including a spinal plate, in the Acanthothoraci) [8], [9]. Given the fact that pelvic spines are known in a number of acanthodians and in one placoderm (spinal plate in the acanthothoracid *Murrindalaspis*, Fig. 7), the presence of the pelvic spine in *Guiyu* and *Psarolepis* (when regarded as stem osteichthyans) can be reasonably explained as a retained primitive feature of gnathostome pelvic girdles (with the assumption of independent loss in chondrichthyans). Similarly, polybasal pelvic fin articulation (observable in *Psarolepis*) can be assumed to be a primitive gnathostome feature based on its distribution in placoderms, chondrichthyans and actinopterygians [49]. However, the combination of pelvic girdle features resembling the placoderm condition (i.e. integrated endoskeletal and dermal elements with large plates as well as the similarity between the pectoral and pelvic girdles) seems difficult to reconcile with scenarios based on prevalent gnathostome phylogenies. It is tempting to consider the pelvic girdle of *Guiyu* and *Psarolepis*, with integrated endoskeletal and dermal components, a retention of the plesiomorphic condition for gnathostomes. For the pectoral girdle, this is the most parsimonious interpretation of the less incongruent distribution of pectoral spines and/or other dermal elements in some placoderms, acanthodians and even chondrichthyans [50]. However, the prevalent phylogenies, which place *Acanthodes* (and the other acanthodids) as stem osteichthyans [12], [14], would favor the interpretation that the placoderm-like pelvic girdle of *Guiyu* and *Psarolepis* is an apomorphic reversal to the plesiomorphy.

Figure 7 represents a simplified scheme showing the distribution of pelvic girdle features among different gnathostome groups, even though we realize that the monophyly of both placoderms and acanthodians is under increasing scrutiny [12], [15] and that the acanthodians, in particular, may well be a paraphyletic assemblage occupying the stem segments of osteichthyans, chondrichthyans and gnathostomes [12].The existence of alternative positions of *Guiyu* and *Psarolepis* (positions I and II in Fig. 7) seems to resonate with other studies that are starting to consider the possibility that some previously identified crown osteichthyans (usually classified as actinopterygians) are actually stem osteichthyans [14].

Friedman and Brazeau [14] used their character scheme to interpret the placement of problematic Silurian-Devonian genera and suggested that several previously identified actinopterygians (e.g. *Ligulalepis* and *Dialipina*) are stem osteichthyans. *Guiyu* and *Psarolepis* possess three of the features listed by Friedman and Brazeau [14] as apomorphies of the total group Sarcopterygii (joint between ethmosphenoid and otoccipital regions of neurocranium, basicranial fenestra, and extensive pore-canal network), yet they also show features not considered by Friedman and Brazeau [14] as justifying membership in the total group Sarcopterygii (e.g. pectoral and pelvic girdle features, buried generations of enamel/odontodes). Given the pelvic girdle features of *Guiyu* and *Psarolepis* described here, it is not inconceivable that *Guiyu* and *Psarolepis* will join other early osteichthyans in populating the most crownward portion of the osteichthyan stem group (position II in Fig. 7) – pending future analyses when more characters become available.

## References

[1] Janvier P (1996). Early Vertebrates.

[2] Janvier P, Arsenault M, Desbiens S (2004). Calcified cartilage in the paired fins of the osteostracan *Escuminaspis laticeps* (Traquair 1880), from the Late Devonian of Miguasha (Québec, Canada), with a consideration of the early evolution of the pectoral fin endoskeleton in vertebrates.. Journal of Vertebrate Paleontology.

[3] Coates MI, Jeffery JE, Ruta M (2002). Fins to limbs: what the fossils say.. Evolution & Development.

[4] Coates MI (2003). The evolution of paired fins.. Theory in Biosciences.

[5] Johanson Z (2010). Evolution of paired fins and the lateral somitic frontier.. Journal of Experimental Zoology Part B: Molecular and Developmental Evolution.

[6] Wake MH (1979). Hyman's comparative vertebrate anatomy. 3rd ed.

[7] Goujet DF (1973). *Sigaspis*, un nouvel arthrodire du Dévonien inférieur du Spitsberg.. Palaeontographica Abt A.

[8] Long JA, Young GC (1988). Acanthothoracid remains from the Early Devonian of New South Wales, including a complete sclerotic capsule and pelvic girdle.. Memoirs of the Association of Australasian Palaeontologists.

[9] Long JA (1997). Ptyctodontid fishes (Vertebrata, Placodermi) from the Late Devonian Gogo Formation, Western Australia, with a revision of the European genus *Ctenurella* Ørvig, 1960.. Geodiversitas.

[10] Zhu M, Yu XB, Choo B, Wang JQ, Jia LT (2012). An antiarch placoderm shows that pelvic girdles arose at the root of jawed vertebrates.. Biology Letters.

[11] Miles RS (1973). Articulated acanthodian fishes from the Old Red Sandstone of England, with a review of the structure and evolution of the acanthodian shoulder-girdle.. Bulletin of the British Museum (Natural History), Geology.

[12] Brazeau MD (2009). The braincase and jaws of a Devonian ‘acanthodian’ and modern gnathostome origins.. Nature.

[13] Maisey JG (2009). The spine-brush complex in symmoriiform sharks (Chondrichthyes; Symmoriiformes), with comments on dorsal fin modularity.. Journal of Vertebrate Paleontology.

[14] Friedman M, Brazeau MD (2010). A reappraisal of the origin and basal radiation of the Osteichthyes.. Journal of Vertebrate Paleontology.

[15] Young GC (2010). Placoderms (armored fish): dominant vertebrates of the Devonian period.. Annual Review of Earth and Planetary Sciences.

[16] Johanson Z (2002). Vascularization of the osteostracan and antiarch (Placodermi) pectoral fin: similarities, and implications for placoderm relationships.. Lethaia.

[17] Young GC (2008). The relationships of antiarchs (Devonian placoderm fishes) – evidence supporting placoderm monophyly.. Journal of Vertebrate Paleontology.

[18] Zhu M, Yu XB, Janvier P (1999). A primitive fossil fish sheds light on the origin of bony fishes.. Nature.

[19] Botella H, Blom H, Dorka M, Ahlberg PE, Janvier P (2007). Jaws and teeth of the earliest bony fishes.. Nature.

[20] Zhu M, Zhao WJ, Jia LT, Lu J, Qiao T (2009). The oldest articulated osteichthyan reveals mosaic gnathostome characters.. Nature.

[21] Basden AM, Young GC, Coates MI, Ritchie A (2000). The most primitive osteichthyan braincase?. Nature.

[22] Schultze H-P, Cumbaa SL, Ahlberg PE (2001). *Dialipina* and the characters of basal actinopterygians.. Major Events in Early Vertebrate Evolution: Palaeontology, Phylogeny, Genetics and Development.

[23] Zhu M, Yu XB, Wang W, Zhao WJ, Jia LT (2006). A primitive fish provides key characters bearing on deep osteichthyan phylogeny.. Nature.

[24] Qiao T, Zhu M (2010). Cranial morphology of the Silurian sarcopterygian *Guiyu oneiros* (Gnathostomata: Osteichthyes).. Science China Earth Sciences.

[25] Zhu M, Schultze H-P (1997). The oldest sarcopterygian fish.. Lethaia.

[26] Yu XB (1998). A new porolepiform-like fish, *Psarolepis romeri*, gen. et sp. nov. (Sarcopterygii, Osteichthyes) from the Lower Devonian of Yunnan, China.. Journal of Vertebrate Paleontology.

[27] Zhu M, Yu XB (2009). Stem sarcopterygians have primitive polybasal fin articulation.. Biology Letters.

[28] Zhu M, Yu XB, Ahlberg PE (2001). A primitive sarcopterygian fish with an eyestalk.. Nature.

[29] Zhu M, Yu XB (2002). A primitive fish close to the common ancestor of tetrapods and lungfish.. Nature.

[30] Friedman M (2007). *Styloichthys* as the oldest coelacanth: implications for early osteichthyan interrelationships.. Journal of Systematic Palaeontology.

[31] Yu X, Zhu M, Zhao WJ (2010). The origin and diversification of osteichthyans and sarcopterygians: rare Chinese fossil findings advance research on key issues of evolution.. Bulletin of the Chinese Academy of Sciences.

[32] Qu QM, Zhu M, Li G (2010). Synchrotron radiation X-ray microtomography reveals the primitive histological architecture of osteichthyan scales. Abstracts of Third International Palaeontological Congress.

[33] Ahlberg PE (1999). Something fishy in the family tree.. Nature.

[34] Chang MM (2000). Fossil fish up for election.. Nature.

[35] Coates MI (2009). Beyond the age of fishes.. Nature.

[36] Schultze H-P (1968). Palaeoniscoidea-Schuppen aus dem Unterdevon Australiens und Kansas und aus dem Mitteldevon Spitzbergens.. Bulletin of the British Museum (Natural History), Geology.

[37] Basden AM, Young GC (2001). A primitive actinopterygian neurocranium from the Early Devonian of southeastern Australia.. Journal of Vertebrate Paleontology.

[38] Gross W (1968). Fragliche Actinopterygier-Schuppen aus dem Silur Gotlands.. Lethaia.

[39] Janvier P (1978). On the oldest known teleostome fish *Andreolepis heder* Gross (Ludlow of Gotland), and the systematic position of the lophosteids.. Eesti NSV Teaduste Akadeemia Toimetised, Geoloogia.

[40] Gross W (1969). *Lophosteus superbus* Pander, ein Teleostome aus dem Silur Oesels.. Lethaia.

[41] Schultze H-P, Märss T (2004). Revisiting *Lophosteus* Pander 1856, a primitive osteichthyan.. The Gross Symposium 2: Advances in Palaeoichthyology.

[42] Zhao WJ, Zhu M (2010). Siluro-Devonian vertebrate biostratigraphy and biogeography of China.. Palaeoworld.

[43] Denison RH (1979). Acanthodii; Schultze H-P, editor.

[44] Gardiner BG (1984). The relationships of the palaeoniscid fishes, a review based on new specimens of *Mimia* and *Moythomasia* from the Upper Devonian of Western Australia.. Bulletin of the British Museum (Natural History), Geology.

[45] Jarvik E (1944). On the exoskeletal shoulder-girdle of teleostomian fishes, with special reference to *Eusthenopteron foordi* Whiteaves.. Kungliga Svenska Vetenskapsakademiens Handlingar.

[46] Stensiö EA (1959). On the pectoral fin and shoulder girdle of the arthrodires.. Kungliga Svenska Vetenskapsakademiens Handlingar.

[47] Forey PL (1980). *Latimeria*: a paradoxical fish.. Proceedings of the Royal Society of London Series B – Biological Sciences.

[48] Gardiner BG (1984). The relationship of placoderms.. Journal of Vertebrate Paleontology.

[49] Rosen DE, Forey PL, Gardiner BG, Patterson C (1981). Lungfishes, tetrapods, paleontology, and plesiomorphy.. Bulletin of the American Museum of Natural History.

[50] Miller RF, Cloutier R, Turner S (2003). The oldest articulated chondrichthyan from the Early Devonian period.. Nature.

